# Activated AXL Ameliorates Alcohol-associated Steatotic Liver Ischemia-Reperfusion Injury by Inhibiting ER stress and Mitochondria-associated Apoptosis

**DOI:** 10.7150/ijbs.103789

**Published:** 2025-01-20

**Authors:** Qi Fang, Qi Yan, Xingyun Liu, Xiaolu Zhang, Lixia Zha, Ruixin Zhang, Zhixin Gao, Jian Du, Lijian Chen

**Affiliations:** 1 Department of Anesthesiology, The First Affiliated Hospital of Anhui Medical University, Hefei 230032, China.; 2 Department of Biochemistry and Molecular Biology, Research Center for Infectious Diseases, School of Basic Medical Sciences, Anhui Medical University, Hefei 230032, China.; 3 Provincial Key Laboratory of Zoonoses of High Institutions in Anhui, Anhui Medical University, Hefei 230032, China.

**Keywords:** Hepatic ischemia-reperfusion injury, AXL, Endoplasmic reticulum stress, Mitochondria, Apoptosis, Alcohol-associated liver disease

## Abstract

Hepatic ischemia-reperfusion (I/R) injury can cause poor prognosis of liver transplantation and hepatectomy, especially in patients with alcohol-associated liver disease (ALD). Apoptosis is closely related to different stages of liver injury, and the death of hepatocytes caused by endoplasmic reticulum (ER) and mitochondria homeostasis perturbation may be key to liver injury. The receptor tyrosine kinases AXL encoded by the gene axl, is a member of the TAM (TYRO3, AXL, and MERTK) family, which participates in various biological processes by binding to the ligand of growth arrest-specific protein 6 (Gas6). However, whether AXL is involved in apoptosis pathways, and the detailed mechanism in hepatic I/R injury remains unknown. In the present study, we found that total AXL is up-regulated while phosphorylated AXL (p-AXL, the active form of AXL) was down-regulated after I/R in human liver tissues from liver transplantation. Consistently, total AXL was found up-regulated while p-AXL was down-regulated during hepatic I/R injury in mice. Pretreatment with Gas6 increased p-AXL expression, reduced ER stress-associated cell apoptosis, alleviated liver damage, and restored ER and mitochondria ultrastructure during hepatic I/R in mice. Furthermore, the ALD model was established by chronic-plus-binge ethanol feeding to explore the role of AXL in I/R liver injury with ethanol-associated steatosis. We found that ALD mice had a lower p-AXL level and were more susceptible to hepatic I/R injury. Importantly, activated AXL ameliorated liver injury by inhibiting IRE1 and PERK pathway to reduce ER stress-associated apoptosis. In conclusion, activated AXL protects alcohol-associated steatotic liver against I/R injury by inhibiting ER stress and mitochondria-associated apoptosis, suggesting that targeting AXL serves as a potential strategy for liver I/R injury, particularly for marginal liver donors with alcohol-associated steatosis.

## 1. Introduction

Hepatic ischemia-reperfusion (I/R) injury is a common clinical complication of liver transplantation (LT), partial hepatectomy and hemorrhagic shock. The underlying mechanisms of hepatic I/R injury are extremely complicated and involved in hepatocytes, Kupffer cells, sinusoidal endothelial cells, neutrophils and others^1^. In recent years, the use of extended criteria donor (ECD) grafts has garnered increasing attention due to the global shortage of liver donors. However, these ECD livers exhibit heightened susceptibility to I/R injury owing to their relatively diminished size or compromised hepatic reserve function^2^, ^3^, such as in the case of a donor with alcohol-associated liver disease (ALD)^4^. Therefore, it is imperative to investigate the underlying mechanism of hepatic I/R injury, and to identify novel drug targets to expand the utilization of donors with alcohol (EtoH)-associated steatosis, a leading cause of chronic liver disease^5^.

Apoptosis is a primary manner of cell death during liver I/R injury^6^. However, the mechanisms underlying hepatocyte apoptosis and the ensuing liver dysfunction are not yet clearly elucidated. The classical apoptosis is mediated by two main pathways. By starting with the activation of cell-surface death receptors, such as Fas, the extrinsic pathway eventually causes the activation of Caspase-8 or 10. The other is the intrinsic pathway, also known as the mitochondrial pathway, which originates from mitochondrial release of cytochrome C and the activation of Caspase-9. Both pathways form signaling cascades that converge on the activation of Caspase-3 which ultimately leads to cell apoptosis. However, Caspase-12 mediates an endoplasmic reticulum (ER)-specific apoptosis pathway, which has been attracting more and more attention. There are three transmembrane ER stress sensors (IRE1, PERK, and ATF6). In non-stressed cells, all three ER stress transducers are kept in an inactive state by binding to the binding immunoglobulin protein (BiP), the ER chaperone, also known as glucose-regulated protein 78KDa (GRP78). ER homeostasis disruption and the accumulation of misfolded proteins accumulate in the ER are referred to as ER stress. Subsequently, three transducers are activated and signals are sent to the nucleus, inhibiting protein synthesis and accelerating misfolded protein degradation to relieve ER stress. This response, in an attempt to restore cellular homeostasis, is called the unfolded protein response (UPR). However, persistent unrelieved ER stress induces apoptosis to remove injured cells by activating the C/EBP-homologous protein (CHOP) and Caspase-12 through terminal UPR signaling^7^. However, the key regulatory molecules of ER stress during liver I/R injury remain unclear.

AXL (also known as ARK, Tyro7 or UFO) belongs to the TAM (TYRO3, AXL, and MERTK) receptor tyrosine kinases, and participates in various biological processes, including immune regulation, cellular signaling, and cancer progression by binding to its primary ligand, the growth arrest-specific protein 6 (Gas6)^8^. AXL consists of extracellular, transmembrane, and intracellular domains. The intracellular section, known as the cytoplasmic tyrosine kinase domain (TKD), contains the KW (I/L) A (I/L) ES consensus sequence, which plays a critical role in tyrosine kinase activity. TAM receptors are activated after receptor dimerization and TKD trans-autophosphorylation upon binding to their extracellular ligands, which subsequently activate intracellular signaling cascades and regulate gene transcription. TAM receptors inhibitors have been in preclinical and clinical development, among those targeting AXL being the most clinically advanced. Our group found that total AXL expression is significantly up-regulated in human liver tissues after the pringle maneuver and reperfusion^9^, and further study demonstrated that AXL activation relieves inflammation during hepatic I/R injury^10^. However, it remains unclear whether AXL plays a role in ER stress during hepatic I/R injury.

In the current study, we found that phosphorylated AXL (p-AXL, the active form of AXL) was down-regulated during liver I/R injury and it predicted the poor prognosis of patients post liver surgery, and activated AXL inhibits ER stress-associated apoptosis to alleviate liver I/R injury. Further study found that ALD mice had a lower p-AXL level and were more susceptible to hepatic I/R injury. Activated AXL ameliorated liver injury by inhibiting IRE1 and PERK pathway to reduce ER stress-associated apoptosis. Our findings provided a potential therapeutic target for marginal liver donors with alcohol-associated steatosis.

## 2. Materials and methods

### 2.1. Human samples

Control liver samples were obtained from patients with hepatic hemangioma undergoing hepatectomy without hepatitis, fatty liver, and drug-induced liver injury. Hepatic I/R injury samples were obtained from patients who had completed LT, and liver biopsies were performed from the left lobe 3 hrs after portal vein reperfusion (before abdominal closure). No upper age limit was set, and no samples from executed prisoners or institutionalized individuals were used in the study. The Institutional Review Board of the First Affiliated Hospital of Anhui Medical University, Anhui Province, China, approved all procedures (No. 20190214), and the study was performed per the principles of the Declaration of Helsinki and local laws. Written informed consent was obtained from the participants or legal representatives of the donors.

### 2.2. Animals

Male C56BL/6J mice (6-8 weeks old) were purchased from Jiangsu Jicui Pharmachem Biotechnology Co. All mice were housed at 25℃ in a 12-hour light-dark cycle with access to food and water *ad libitum*. The animal experiments were approved by the Animal Ethics Committee of Anhui Medical University (License No: LLSC20190022).

### 2.3. The hepatic I/R model

Intraperitoneal injection of rmGas6 (5 µg/mouse), (R&D Systems, 8310-GS; America), vehicle, and R428 (125 mg/kg) (MCE, HY-15150; America) were performed 2 h before I/R injury. The animals were randomly assigned to six groups, including the sham-operated, I/R, I/R + vehicle, I/R + rmGas6, I/R + R428, and I/R + rmGas6 + R428 groups (each group n = 6)^10^. All mice were anesthetized with an intraperitoneal injection of sodium pentobarbital (0.3%, 50 mg/kg). A stable model of local warm hepatic ischemia was established as previously mentioned. The same abdominal incision was made on mice in the sham-operated group, while their blood vessels were not clamped. The reperfusion time differed after 60 min of ischemia. The mice were euthanized with an overdose of intraperitoneal injection of sodium pentobarbital (0.3%, 150 mg/kg) after surgery and their serum and liver samples were collected immediately for analysis.

### 2.4. The ALD model

Lieber-DeCarli-EtOH diets, isocaloric pair-matched liquid control diets, and maltose-dextrin were purchased from Trophic Animal Feed High-Tech Co.,Ltd (China). The fresh liquid diet was prepared daily and 95% ethanol was added immediately before feeding.

Mice were initially fed the control Lieber-DeCarli diet ad libitum for 5 d to acclimatize them to liquid diets and tube feeding. Afterward, mice in the EtOH-fed group were allowed free access to the Lieber-DeCarli-EtOH diet containing 5% (vol/vol) ethanol for 10 d (ALD group), and those in the control group were pair-fed with the isocaloric pair-matched liquid control diets (Pair group). Early in the morning of day 11, EtOH-fed and pair-fed mice received a single gavage of EtOH (5 g/kg) or isocaloric maltose-dextrin (9 g/kg). They were euthanized 9 h later^11^. The serum and liver samples were collected immediately for analysis. Mice required the pretreatment with rmGas6/R428 referred in the previous section.

### 2.5. Primary hepatocytes isolation

The livers of mice were perfused with Ca^2+^ and Mg^2+^-free Hank's buffered salt solution containing EGTA (2.5 mM) via the portal vein, and the inferior vena cava was severed, followed by perfusion with 0.05% collagenase IV HBSS solution. The digested livers were dissected without their gallbladders, after which they were gently teased with forceps until sediment-like material entered the solution. The resulting cell suspensions were filtered through a 100 μm nylon cell strainer. The hepatocytes were separated from the non-parenchymal cells by one cycle of differential centrifugation (50 g/min for 5 min). The bottom hepatocyte pellet was resuspended and centrifuged using 50% percoll for 10 min at 1400 rpm/min. The resulting cell pellets were washed with DMEM supplemented with 10% FBS, and the cells were seeded into collagen pre-coated 100 mm tissue plates.

### 2.6. The OGD/R model of primary hepatocytes and cell death assays

Primary hepatocytes were isolated according to the method described above. rmGas6 or R428 was used for pretreatment 2 h before OGD/R. The specific experimental groups were as follows: control, OGD/R (6 h), OGD/R (6 h) + vehicle, OGD/R (6 h) + rmGas6 (200 ng/mL), OGD/R (6 h) + R428 (200 nM), OGD/R (6 h) + rmGas6 (200 ng/mL) + R428 (200 nM)^10^.

Cells were digested with trypsin and collected. As per the manufacturer's protocol, the cells were stained with Annexin V-FITC and propidium iodide for 15 min (BD Biosciences, C1062M). The cells were placed on ice and protected from light, and the Beckman Coulter Cytoflex was used for analysis.

### 2.7. Liver function analysis

The mice were anesthetized and executed to collect the blood samples after hepatic I/R surgery, and stood at room temperature for 2 h. The samples were then centrifuged at 3500 rpm/min for 10 min at 4°C. According to the instructions of ALT and AST kits (Nanjing Jiancheng Institute of Biological Engineering, China), the absorbance was measured at 405 nm and the ALT and AST contents were calculated respectively.

### 2.8. Liver histology and immunohistochemistry (IHC)

Liver tissues were collected and fixed with 10% formalin, and then sliced into 4 µm thick sections. Hematoxylin and eosin staining was used to detect the percentage of liver necrosis after hepatic I/R injury. Oil red O staining was used to detect lipid droplets in the liver. According to the manufacturer's instructions, cleaved-Caspase-3 (ImmunoWay, USA; 1:100), CHOP (Affinity Biosciences, China; 1:100) and p-AXL (CST, USA; 1:100) were stained for IHC in paraffin sections.

### 2.9. Western blotting

Protein extracts were obtained by homogenizing samples in a cell lysis buffer. Equal amounts of protein samples were loaded on 10% polyacrylamide gels, separated by SDS-PAGE, and then transferred to nitrocellulose membranes, and incubated with primary antibodies specific AXL (CST, USA; 1:1000), p-AXL (CST, USA; 1:1000), Bax (Proteintech, China; 1:3000), cleaved-Caspase-3 (ImmunoWay, USA; 1:1000), Bcl-2 (Wanlei, China; 1:1000), CHOP (Affinity Biosciences, China, 1:1000), BiP (CST, USA; 1:1000), PERK (Affinity Biosciences, China, 1:1000), p-PERK (Affinity Biosciences, China, 1:1000), IRE1 (Wanlei, China; 1:1000), p-IRE1 (Wanlei, China; 1:1000), XBP1s (Proteintech, China; 1:1000), ATF4 (Proteintech, China; 1:1000), cleaved-Caspase-12 (Wanlei, China; 1:1000), and cleaved-Caspase-9 (Proteintech, China; 1:1000) at 4 °C overnight. The detection of phosphorylated proteins is the same as that of other proteins. Occasionally, samples were run on parallel gels and probed separately. Bands were analyzed by ImageJ software.

### 2.10. Transmission electron microscopy (TEM)

According to the above grouping, the livers of mice after I/R surgery or primary hepatocytes were extracted and inoculated on 6-well plates and then treated with OGD/R. Tissue and cells were fixed with 2.5% glutaraldehyde at 4 ℃ overnight. And then rinsed, stained, dehydrated, permeated with 100% acetone, embedded and polymerisated. The samples were observed by TEM.

### 2.11. Statistical analysis

GraphPad Prism 8 software was used for all statistical analyses. Quantitative data are presented as the mean ± standard error of the mean. The one-way analysis of variance and the independent t-test were performed to compare the means of different values. *P* < 0.05 was considered statistically significant.

## 3. Results

### 3.1 The phosphorylation of hepatic AXL is down-regulated during I/R injury

**Figure 1:**
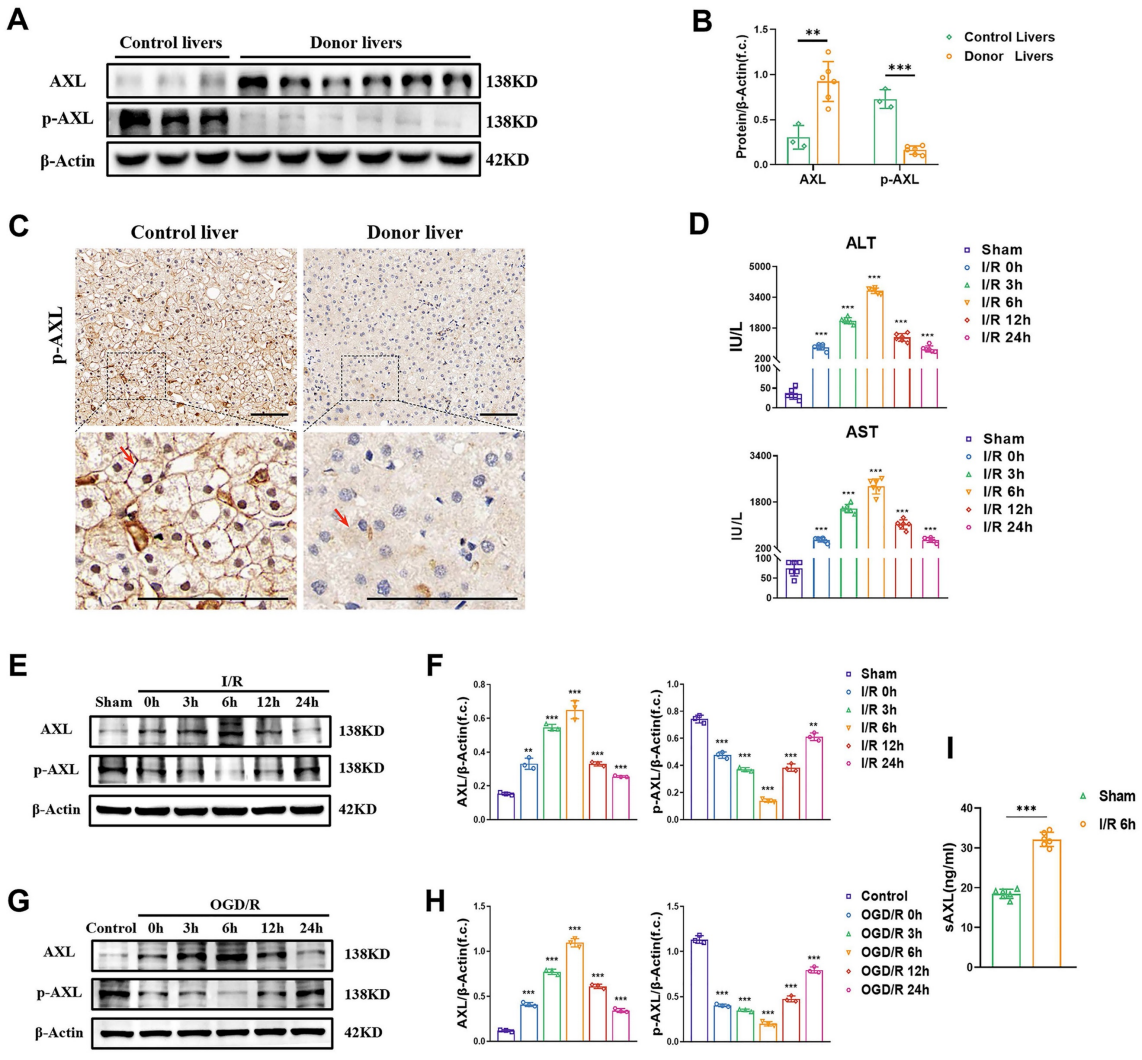
** The phosphorylation of hepatic AXL is down-regulated during I/R injury. (A-B)** Representative relative protein expression of AXL and p-AXL in control liver tissues (n = 3) and transplant donor liver tissues (n = 6) were detected by Western blotting (WB) analysis and densitometric quantification. **(C)** Representative liver biopsies from transplant donors, performed from the left lobe 3 h after portal vein reperfusion, and from healthy control individuals where p-AXL expression (indicated by red arrows) was determined by immunohistochemical staining. Scale bar = 100 µm. **(D)** Serum ALT and AST levels in mice with sham operation or at 0, 3, 6 ,12, 24 h after I/R (each group n = 6). **(E-F)** WB analysis of the relative protein expression of AXL and p-AXL in mice liver tissues with sham operation or at 0, 3, 6, 12, 24 h after I/R and densitometric quantification. **(G-H)** WB analysis of the relative protein expression of AXL and p-AXL in primary mouse hepatocytes at 0, 3, 6, 12, 24 h after OGD/R and densitometric quantification. **(I)** Serum sAXL level in mice with sham operation or 6 hrs after I/R (each group n = 6). Data are shown in mean ± SD. **P < 0.01, ***P < 0.001; ALT, alanine aminotransferase; AST, aspartate aminotransferase; p-AXL, phosphorylated AXL; sAXL, soluble AXL; I/R, ischaemia /reperfusion; OGD/R, oxygen-glucose deprivation/reoxygenation.

To investigate whether total AXL/p-AXL are dysregulated under surgical ischemic conditions, and whether they are involved in hepatic I/R injury in humans, we examined their expressions in liver biopsies from donor grafts and in control individuals. Our results showed that total AXL expression levels were higher in donor livers than in livers from control individuals, whereas the expression trend of p-AXL was reversed (Figure 1A-C). To explore the variation tendency of total AXL/p-AXL during hepatic I/R, we constructed murine models with different time of reperfusion after 60 min of ischemia. The serum levels of liver enzymes (ALT/AST) in mice gradually increased until 6 h, and then gradually recovered with time after I/R injury (Figure 1D). Similarly, total AXL expression also peaked at 6 h and then gradually returned to the basal level. In this process, the expression of p-AXL was lowest at 6 h (Figure 1E&F). Meanwhile, we performed OGD/R treatment on primary hepatocytes *in vitro*, and total AXL/p-AXL showed the same trend observed during hepatic I/R in mice (Figure 1G&H). Impressively, we detected soluble AXL (sAXL) in the serum of liver I/R mice and found that sAXL was greatly elevated. Soluble AXL is a cleavage product of total AXL without physiological activity, but it can competitively block AXL receptor. This result provided an explanation for the increase in total AXL and decrease in p-AXL during hepatic I/R (Figure 1I). Therefore, the phosphorylation of AXL is decreased during hepatic I/R.

### 3.2 Activated AXL protects against hepatic I/R injury by inhibiting apoptosis

**Figure 2:**
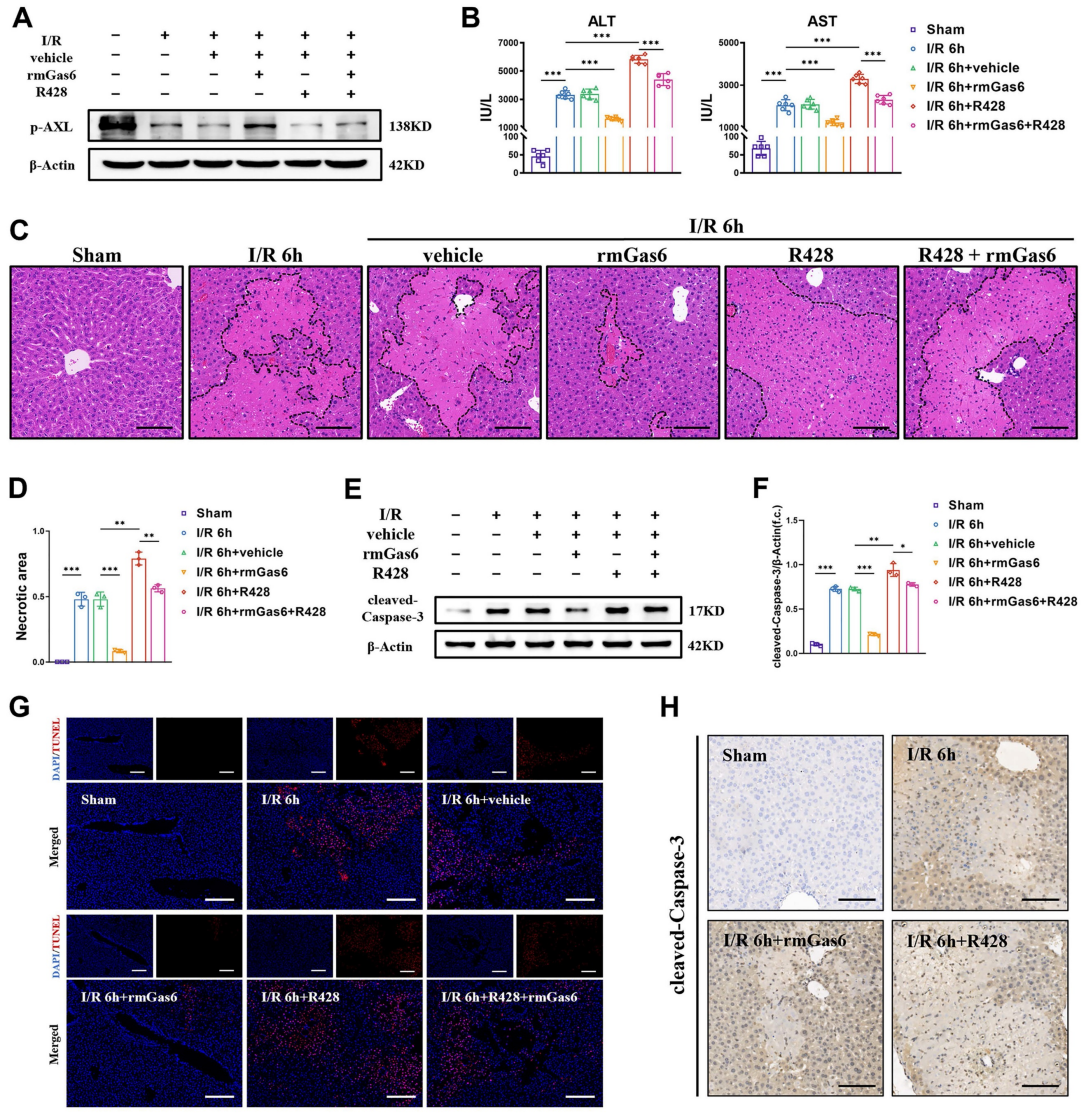
** Activated AXL protects against hepatic I/R injury by inhibiting apoptosis. (A)**WB analysis of the relative protein expression of p-AXL in mice liver tissues of control group and treatment group (as shown in the figure) after liver I/R.** (B)** Serum ALT and AST levels in mice (each group n = 6). **(C-D)** The necrosis area of liver tissues in mice were shown by H&E staining analysis and quantification. Scale bar = 100 μm. **(E-F)** WB analysis of the relative protein expression of cleaved-Caspase3 in mice and densitometric quantification. **(G)** Liver sections were analysed by TUNEL staining for the level of cell apoptosis in mice. Scale bar = 200 µm. **(H)** Liver sections immunohistochemical staining for cleaved-Caspase-3 in mice. Scale bar = 100 µm. Data are shown in mean ± SD. *P < 0.05, **P < 0.01, ***P < 0.001; ALT, alanine aminotransferase; AST, aspartate aminotransferase; p-AXL, phosphorylated AXL; rmGas6, recombinant growth arrest-specific protein 6; I/R, ischaemia/reperfusion; H&E, hematoxylin and eosin; TUNEL, Terminal deoxynucleotidyl transferase-mediated nick end labeling.

To explore whether AXL inhibition leads to I/R injury and the detailed mechanism, we used exogenous rmGas6 to activate AXL with intraperitoneal injection. In line with the findings in human samples, the expression of p-AXL was decreased in liver I/R mice. When AXL was activated with rmGas6, the increase of p-AXL level indicated that AXL was activated (Figure 2A). Hepatic I/R injury caused liver damage and elevated serum levels of ALT and AST compared with the sham-operated group, and rmGas6 injection reversed these effects. Consistently, R428, a specific inhibitor of AXL, significantly exacerbated the area of liver necrosis and elevated serum levels of ALT and AST (Figure 2B-D). Moreover, the level of pro-apoptotic protein cleaved-Caspase-3 (the active form of Caspase-3) were elevated in liver I/R mice, pretreatment with rmGas6 reversed these trends. TUNEL assay and immunohistochemical staining with cleaved-Caspase-3 of liver tissue sections also confirmed that activated AXL protected liver against I/R injury by inhibiting apoptosis *in vivo* (Figure 2E-H).

Next, we further conducted the experiments *in vitro*. We isolated primary hepatocytes by in situ perfusion of liver and then subjected them to oxygen-glucose deprivation reperfusion (OGD/R). Similarly, a substantial recovery of p-AXL reduction and the expression of cleaved-Caspase-3 was reversed during OGD/R were observed in primary hepatocytes pretreated with rmGas6 (Figure S1A&B). Furthermore, a flow cytometry assay and TUNEL assays confirmed that rmGas6 pretreatment reduced OGD/R-induced hepatocyte apoptosis (Figure S1C-E). These data indicated that the activation of AXL protected liver from I/R injury by inhibiting apoptosis.

### 3.3 Activated AXL attenuates ER stress and mitochondria-associated apoptosis during liver I/R injury

**Figure 3:**
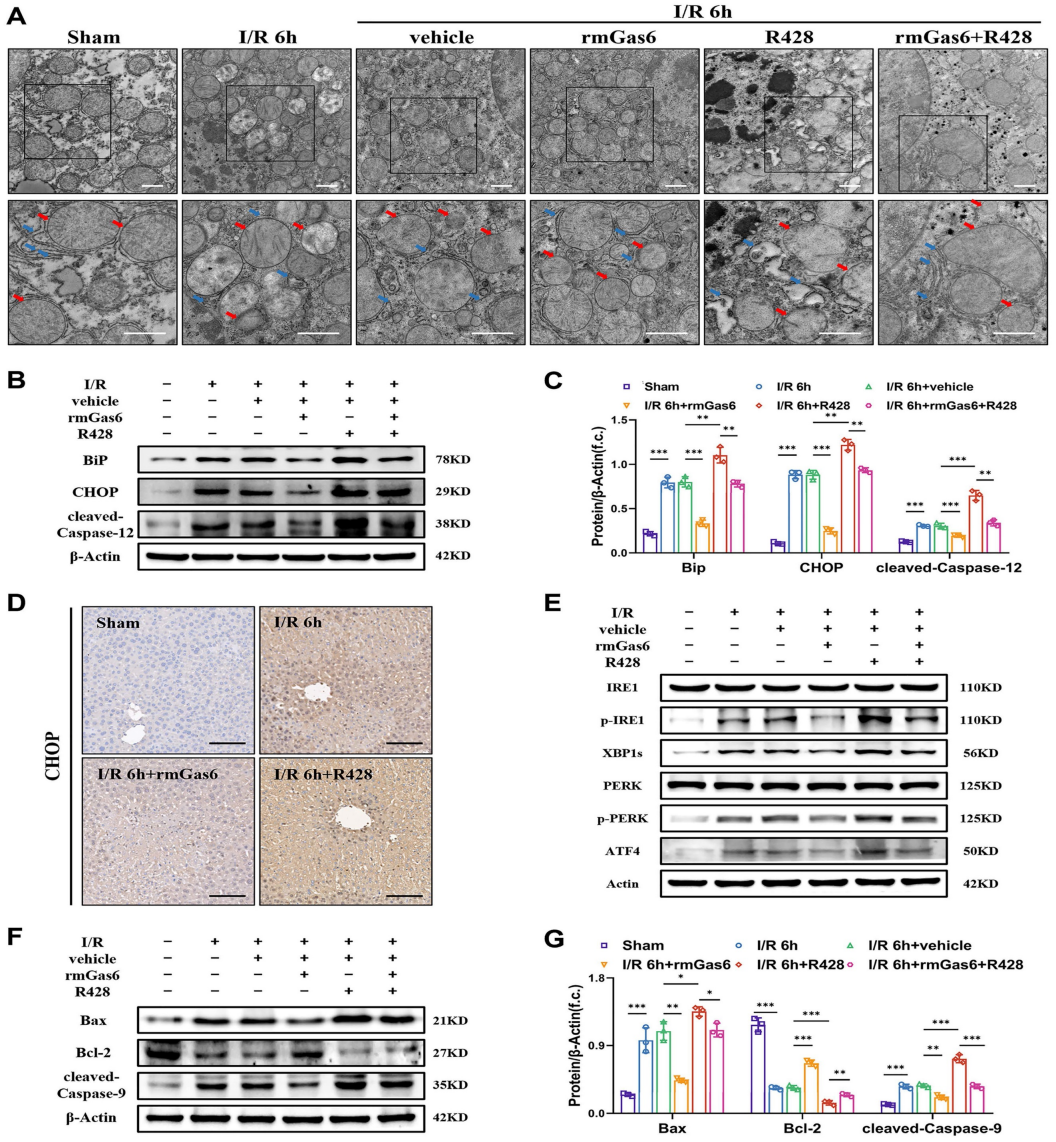
** Activated AXL attenuates ER stress and mitochondria-associated apoptosis during liver I/R injury. (A)** Mice liver tissues ultrastructural of control group and treatment group (as shown in the figure) after liver I/R viewed by TEM. Red arrows indicate mitochondria. Blue arrows indicate endoplasmic reticulum. Scale bar = 1 µm.** (B-C)** WB analysis of the relative protein expression of BiP, CHOP and cleaved-Caspase-12 in mice and densitometric quantification.** (D)** Liver sections immunohistochemical staining for CHOP in mice. Scale bar = 100 µm. **(E)** WB analysis of the relative protein expression of IRE1, p-IRE1, XBP1s, PERK, p-PERK and ATF4 in mice.** (F-G)** WB analysis of the relative protein expression of Bax, Bcl-2 and cleaved-Caspase-9 in mice and densitometric quantification. Data are shown in mean ± SD. *P < 0.05, **P < 0.01, ***P < 0.001; I/R, ischaemia/reperfusion; rmGas6, recombinant growth arrest-specific protein 6.

To further investigate whether AXL affects ER during liver I/R, the ultrastructure of liver tissues was detected by TEM. We found that ER was abnormal, with dilatation, vacuolization and the disappearance of sheet folded tubular structure after I/R. The damage was alleviated in the rmGas6 pretreatment groups with a relatively intact ER structure, while R428 exacerbated IR-induced damage (Figure 3A). The Western blotting results showed that BiP, CHOP and cleaved-Caspase-12 (the active form of Caspase-12) levels were upregulated, suggesting that hepatic I/R leads to ER stress and its associated apoptosis. However, the rmGas6 pretreatment reversed these effects, and R428 pretreatment aggravated IR-induced excessive ER stress and cell apoptosis (Figure 3B&C). The immunohistochemistry of liver tissues with CHOP staining also indicated rmGas6 pretreatment alleviated liver injury by inhibiting CHOP up-regulation (Figure 3D). In eukaryotic cells, three ER trans-membrane proteins mediate the canonical UPR: the two kinases, IRE1and PERK, and the transcription factor precursor ATF6. If the stress is prolonged, or the adaptive response fails, apoptotic cell death ensues through activation of Caspase-12 and CHOP, *et al.* As for the apoptosis effect, the first two kinases, IRE1and PERK, are mainly involved^12^, ^13^. Western blotting analysis found that hepatic I/R increased the levels of phosphorylated-IRE1 (p-IRE1, the active form of IRE1) and its downstream effector XBP1s, phosphorylated-PERK (p-PERK, the active form of PERK) and its downstream ATF4, and all these trends were reversed by rmGas6 pretreatment (Figure 3E). Thus, these findings indicated that activated AXL inhibits IRE1 and PERK branches of UPR in ER stress to protect against hepatic I/R injury. Of note, changes in ultrastructure observed by TEM also indicated mitochondria swelling and cristae vague after I/R, and activated AXL also significantly restored the mitochondrial ultrastructure (Figure 3A). As a classical pathway of endogenous apoptosis, we also investigated whether p-AXL plays any role in mitochondria-related apoptosis. The results showed that activated AXL inhibited the level of cleaved-Caspase-9 (the active form of Caspase-9) and Bax while increased the expression of Bcl-2 during liver I/R injury in mice (Figure 3F&G). Together, all these confirmed that AXL activation attenuated ER stress and mitochondria-associated apoptosis during liver I/R injury.

### 3.4 AXL activation alleviates ER stress and mitochondria-associated hepatocytes apoptosis during OGD/R *in vitro*

**Figure 4:**
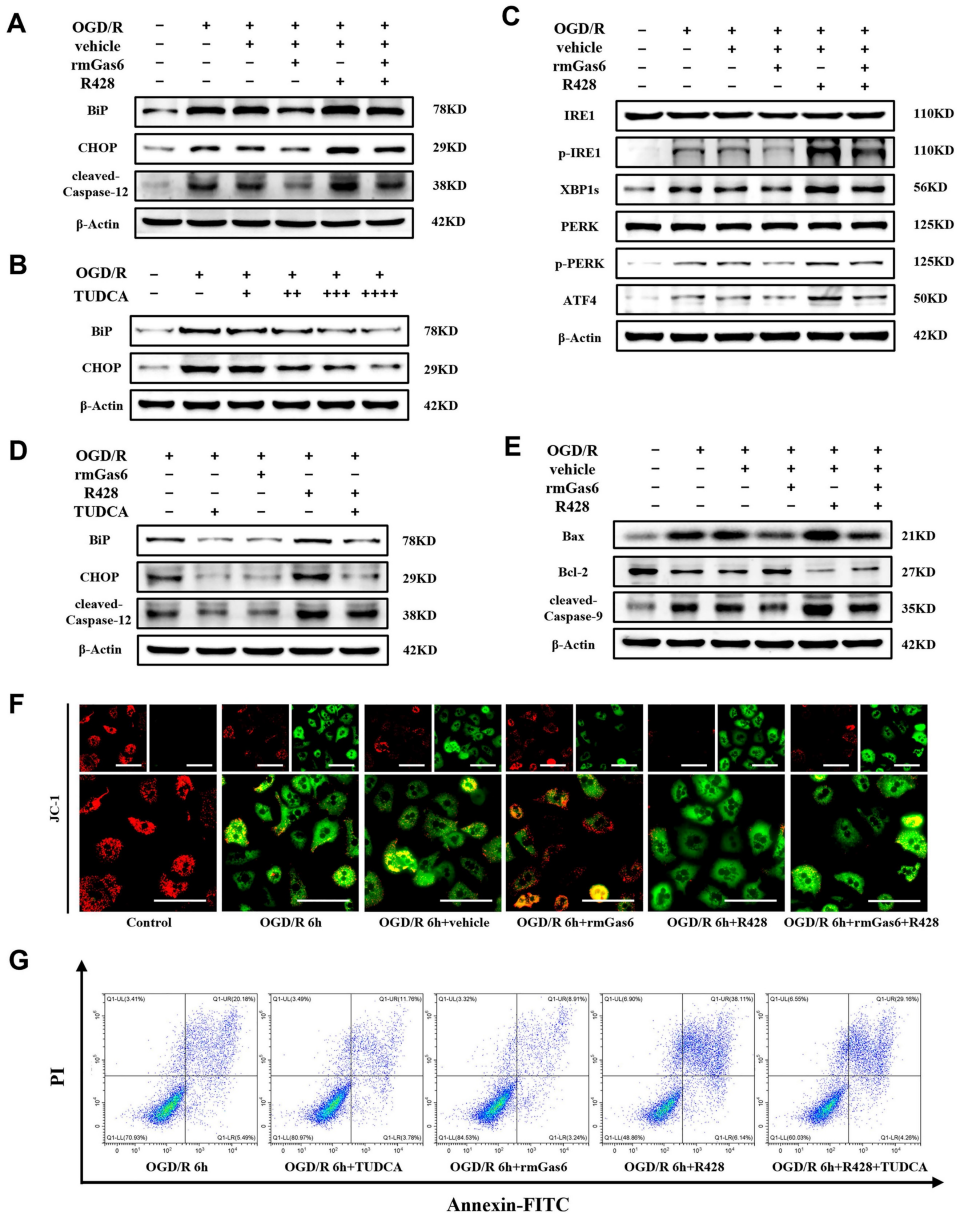
** AXL activation alleviates ER stress and mitochondria-associated hepatocytes apoptosis during OGD/R *in vitro*. (A)** WB analysis of the relative protein expression of BiP, CHOP and cleaved-Caspase-12 in primary mouse hepatocytes of control group and treatment group (as shown in the figure) after OGD/R.** (B)** WB analysis of the relative protein expression of BiP and CHOP in primary mouse after OGD/R with TUDCA pretreatment (**+** = 100 µM, **++** = 200 µM, **+++** = 300 µM, **+++** = 400 µM). **(C)** WB analysis of the relative protein expression of IRE1, p-IRE1, XBP1s, PERK, p-PERK and ATF4 in primary mouse hepatocytes of control group and treatment group (as shown in the figure) after OGD/R. **(D)**WB analysis of the relative protein expression of BiP, CHOP and cleaved-Caspase-12 in primary mouse hepatocytes after OGD/R with rmGas6/R428/TUDCA (200 µM) pretreatment. **(E)** WB analysis of the relative protein expression of Bax, Bcl-2 and cleaved-Caspase-9 in primary mouse hepatocytes of control group and treatment group (as shown in the figure) after OGD/R. **(F)** Immunofluorescence staining of JC-1 showed the mitochondrial membrane potential in primary hepatocytes. Red fluorescence indicates JC-1 aggregates, and green fluorescence indicates JC-1 monomers. Scale bar = 100 µm.** (G)** Flow cytometry assay showing the apoptosis level of primary mouse hepatocytes. OGD/R, oxygen-glucose deprivation/reoxygenation; rmGas6, recombinant growth arrest-specific protein 6; TUDCA, tauro ursodeoxycholic acid.

Consistent with the experiments *in vivo* presented in Figure 3, ER stress induction by OGD/R resulted in terminal UPR signaling (e.g., BiP, CHOP and Caspase-12) responses and could be attenuated by rmGas6 pretreatment *in vitro* (Figure 4A). Tauro ursodeoxycholic acid (TUDCA), a well-established inhibitor of ER stress, sufficiently blocked ER stress responses in OGD/R-treated hepatocytes in a dose dependent manner (Figure 4B). As expected, AXL activated with rmGas6 significantly inhibited the levels of p-IRE1, XBP1s, p-PERK, and ATF4 in OGD/R-treated primary hepatocytes *in vitro* (Figure 4C). Pretreatment with rmGas6 was similar to the effect of TUDCA, and TUDCA application reversed the deleterious effects of R428 (Figure 4D). Additionally, rmGas6 pretreatment hindered OGD/R-induced mitochondria-associated hepatocytes apoptosis by decreasing the expression of cleaved-Caspase-9 and Bax while increasing the level of Bcl-2 during OGD/R *in vitro* (Figure 4E). Besides, immunofluorescence staining of JC-1 in primary hepatocytes also suggested a reduction in mitochondrial membrane potential (MMP) during OGD/R, evident by a decline in JC-1 aggregates (red fluorescence) and an increase in JC-1 monomers (green fluorescence). Notably, rmGas6 pretreatment reinstated MMP, also indicating that activated AXL mitigated mitochondria-associated apoptosis during hepatic I/R injury (Figure 4F). Furthermore, flow cytometry assay confirmed that rmGas6 and TUDCA pretreatment both reduced OGD/R-induced apoptosis (Figure 4G). Together, these results further validated that p-AXL inhibited ER stress and mitochondria-associated apoptosis during OGD/R in primary hepatocytes.

### 3.5 Phosphorylated AXL is downregulated and the susceptibility to I/R injury is increased in liver of ALD mice

**Figure 5:**
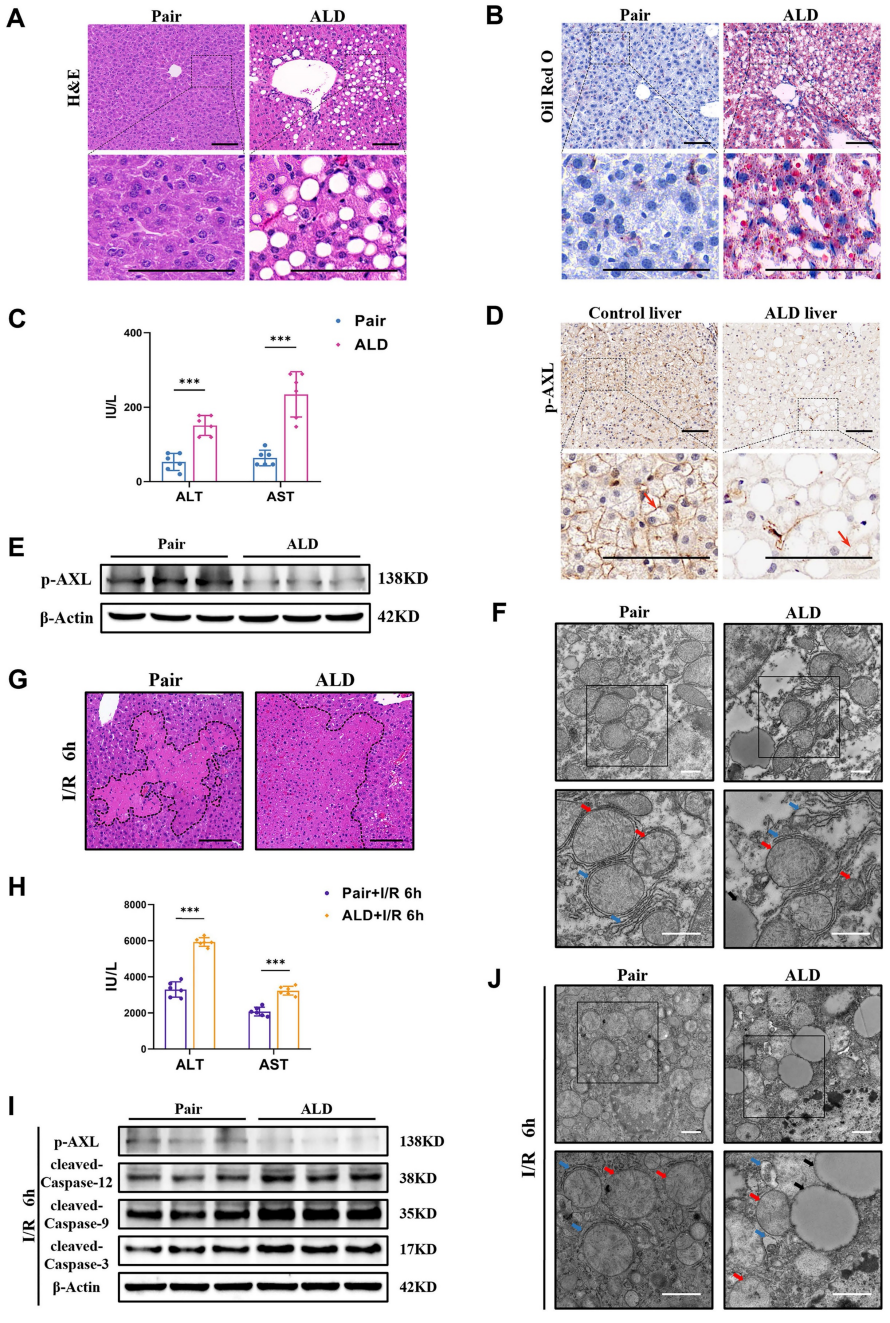
** Phosphorylated AXL is down-regulated and the susceptibility to I/R injury is increased in liver of ALD mice. (A)** H&E staining of the liver sections in Pair and ALD mice. Scale bar = 100 µm.** (B)** Oil red O staining of the liver sections in Pair and ALD mice. Scale bar = 100 µm.** (C)** Serum ALT and AST levels in Pair and ALD mice (each group n = 6).** (D)** Liver biopsies from healthy control individuals and end-stage liver disease patients caused by alcohol consumption where p-AXL expression (indicated by red arrows) was determined by immunohistochemical staining. Scale bar = 100 µm. **(E)** WB analysis of the relative protein expression of p-AXL in Pair and ALD mice.** (F)** Liver tissue ultrastructural of Pair and ALD mice viewed by TEM. Red arrows indicate mitochondria. Blue arrows indicate endoplasmic reticulum. Black arrows indicate lipid droplets. Scale bar = 1 µm.** (G)** The necrosis area of liver tissues in Pair and ALD mice after I/R were shown by H&E staining analysis. Scale bar = 100 μm.** (H)** Serum ALT and AST levels in Pair and ALD mice after liver I/R (each group n = 6).** (I)** WB analysis of the relative protein expression of p-AXL, cleaved-Caspase-12, cleaved-Caspase-9 and cleaved-Caspase-3 in Pair and ALD mice after liver I/R.** (J)** Liver tissue ultrastructural of Pair and ALD mice after I/R viewed by TEM. Red arrows indicate mitochondria. Blue arrows indicate endoplasmic reticulum. Black arrows indicate lipid droplets. Scale bar = 1 µm. **P < 0.01, ***P < 0.001; ALD, alcoholic liver disease; ALT, alanine aminotransferase; AST, aspartate aminotransferase; p-AXL, phosphorylated AXL; I/R, ischaemia/reperfusion; H&E, hematoxylin and eosin; TEM, Transmission electron microscope.

Some patients undergoing liver resection or donors with ALD have a higher risk of post-hepatectomy liver failure as both conditions exacerbate I/R injury. Therefore, we used chronic plus binge alcohol (Gao-binge) feeding followed by I/R surgery to investigate liver I/R injury with ethanol-associated steatosis (Figure S2A). Macroscopic yellowing, enlargement and tortoiseshell pattern detail of liver in ALD mice were observed (Figure S2B). Histopathological studies showed that there were marked microsteatosis and macrosteatosis, as well as hepatocytes ballooning, in ALD mice (ALD group) compared with isocaloric paired feeding mice (Pair group) (Figure 5A). Oil Red O staining also confirmed severe hepatic steatosis and lipid accumulation in ALD mice (Figure 5B). There were also elevated serum levels of ALT/AST in ALD mice (Figure 5C). The expression of p-AXL was down-regulated by immunohistochemical staining in livers obtained from patients with the end-stage liver disease caused by massive and chronic alcohol consumption (Figure 5D). Western blotting was confirmed that p-AXL level was down-regulated in the liver of ALD mice (Figure 5E), as well as in primary hepatocytes treated with EtOH (Figure S2C). Moreover, TEM showed lipid droplets, relatively dilated ER lumen, swollen mitochondria and cristae vague in the liver of ALD mice compared with the Pair group (Figure 5F). More importantly, ALD mice showed more severe liver damage after I/R with larger necrotic areas and higher serum transaminase levels (ALT/AST) compared with the Pair group (Figure 5G&H). Also, compared with the Pair group, the level of p-AXL was down-regulated while the expression levels of cleaved-Caspase-12, cleaved-Caspase-9 and cleaved-Caspase-3 were significantly up-regulated after hepatic I/R treatment in ALD mice (Figure 5I). TEM showed more serious ER dilatation, swollen mitochondria and vacuolar degeneration in the livers of ALD mice compared with the Pair group (Figure 5J). Together, these studies strongly supported that alcohol-associated liver disease induced ER stress and mitochondria-associated apoptosis and increased the susceptibility of mouse liver to I/R injury, and p-AXL may be involved in the pathogenesis.

### 3.6 AXL activation attenuates hepatic I/R injury by reducing ER stress and mitochondria- associated apoptosis in ALD mice

**Figure 6:**
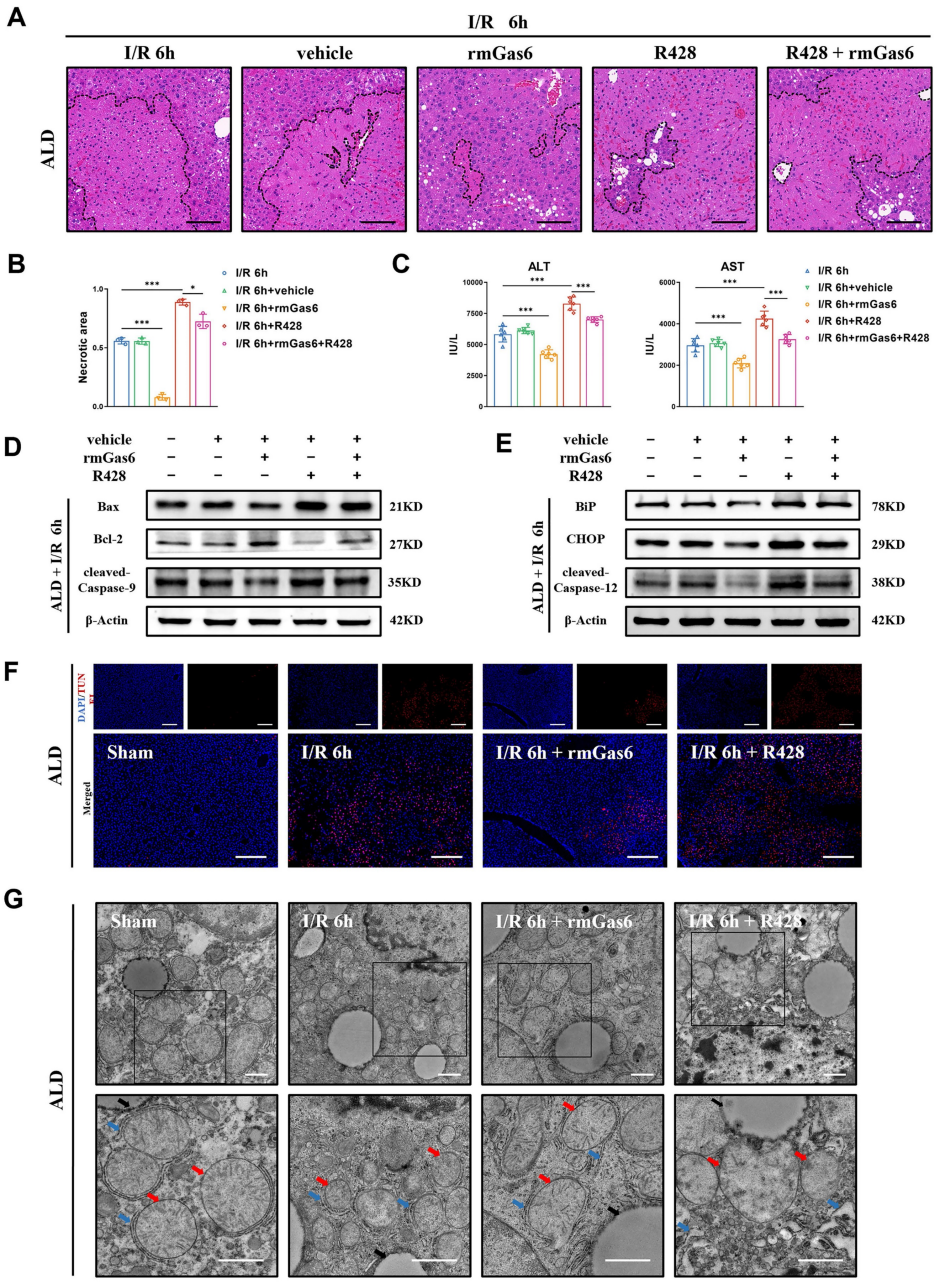
** Activated AXL attenuates hepatic I/R injury by reducing ER stress and mitochondria-associated apoptosis in ALD mice. (A-B)** The necrosis area of liver tissues in ALD mice after I/R with rmGas6/R428 pretreatment were shown by H&E staining analysis and quantification. Scale bar = 100 μm. **(C)** Serum ALT and AST levels in ALD mice after liver I/R with rmGas6/R428 pretreatment (each group n = 6). **(D-E)** WB analysis of the relative protein expression of Bax, Bcl-2, cleaved-Caspase-9, BiP, CHOP and cleaved-Caspase-12 in ALD mice after liver I/R with rmGas6/R428 pretreatment. **(F)** Liver sections of control group and treatment group (as shown in the figure) ALD mice after I/R were analysed by TUNEL staining for the level of cell apoptosis. Scale bar = 200 µm.** (G)** Liver tissue ultrastructural of control group and treatment group (as shown in the figure) ALD mice after I/R viewed by TEM. Red arrows indicate mitochondria. Blue arrows indicate endoplasmic reticulum. Black arrows indicate lipid droplets. Scale bar = 1 µm. *P < 0.05, **P < 0.01, ***P < 0.001; ALD, alcoholic liver disease; I/R, ischaemia/ reperfusion; ALT, alanine aminotransferase; AST, aspartate aminotransferase; rmGas6, recombinant growth arrest-specific protein 6; H&E, hematoxylin and eosin; TEM, Transmission electron microscope; TUNEL, Terminal deoxynucleotidyl transferase-mediated nick end labeling.

As mentioned above, the ALD mice suffered from more serious hepatic I/R injury through ER stress and mitochondria-associated apoptosis. Next, we investigated whether activated AXL also has any protective impact on I/R injury in ALD mice. Pretreatment with rmGas6 attenuated IR-induced liver injury as evidenced by the reduced necrotic area (Figure 6A&B), and decreased serum transaminase levels (ALT/AST) (Figure 6C). Furthermore, pretreatment with rmGas6 attenuated mitochondria-associated apoptosis during liver I/R injury in ALD mice (Figure 6D), and also reduced ER stress-associated apoptosis (Figure 6E). Moreover, TUNEL also showed that AXL activation reduced IR-induced apoptosis in mouse liver tissues (Figure 6F). Besides, TEM showed visually that rmGas6 pretreatment could help ER and mitochondria recover from hepatic I/R injury. On the contrary, inhibition AXL with R428 greatly aggravated liver I/R injury by worsening ER dilation and apoptosis (Figure 6G). Taken together, these studies provided experimental evidence for the protective role of activated AXL in inhibiting ER stress and mitochondria-associated apoptosis during liver I/R injury in ALD mice.

## 4. Discussion

**Figure 7:**
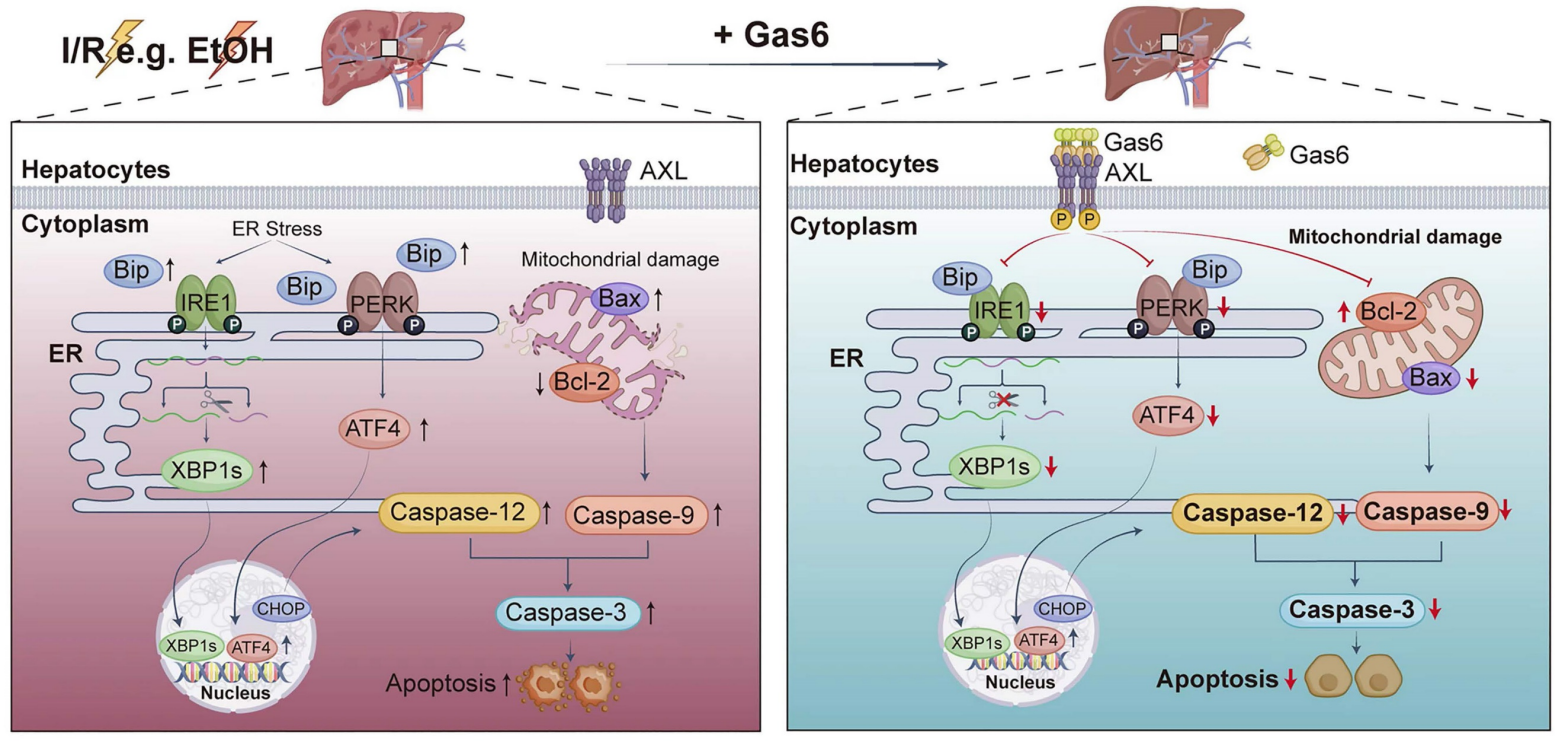
** Schematic drawing summarizes protective effect of activated AXL against alcoholic fatty liver ischemia-reperfusion injury by inhibiting ER stress and mitochondria-related hepatocytes apoptosis. Hepatic I/R injury and EtOH induce hepatocytes apoptosis.** Meanwhile, activated AXL expression is down-regulated. IRE1 and PERK pathways are involved in hepatocytes apoptosis during hepatic I/R injury. Activated AXL inhibits ER stress related apoptosis during hepatic I/R injury. Meanwhile, p-AXL exerts a protective role partly through mitochondria-related apoptosis. More importantly, the molecular mechanism still presents in ALD mice.

This study explored the role of AXL in regulating ER stress and mitochondria-associated apoptosis during hepatic I/R injury. Our results demonstrated that Gas6 pretreatment activated AXL and reduced ER stress and mitochondria-associated hepatocytes apoptosis during hepatic I/R *via* IRE1/XBP1 and PERK/ATF4 pathways, as was in ALD mice (Figure 7). Our previous study demonstrated a decrease of serum Gas6 level and an increase of total AXL during hepatic I/R^10^. However, the active form of AXL (p-AXL) exerting physiological activity is down-regulated. In the present study, we found that soluble AXL (sAXL) was greatly elevated in the serum of liver I/R mice. Some metalloproteinases such as ADAM10/17 cut the extracellular domain of AXL and release into blood in the form of soluble AXL, which was confirmed to be an accurate biomarker of advanced liver fibrosis, cirrhosis and HCC^14^. Soluble AXL also can competitively combine with the ligand Gas6 but has no physiological activity^15^. To the best of our knowledge, our study demonstrated for the first time that serum sAXL is elevated during liver I/R, providing an alternative explanation for the increase in total AXL and decrease in p-AXL during hepatic I/R. Inhibiting activity of metalloproteinases to reduce the exfoliation of AXL's extracellular region may be a new target for the treatment of hepatic I/R injury.

Hepatic I/R injury is a local aseptic inflammatory response driven by innate immunity; however, the exact mechanisms of cell death during the process remain unclear. These mechanisms can be classified as inflammatory (necrosis, pyroptosis and ferroptosis) and non-inflammatory (apoptosis), depending on whether damage-associated molecular patterns are released^16^. ER stress has been linked to several phases of liver injury, and ER stress-mediated hepatocyte death may be the key underlying mechanism of liver injury^17^. I/R injury stimulates ER stress, which can lead to the accumulation of unfolded proteins, destroy ER homeostasis, and eventually lead to cell apoptosis^18^. Increasing evidence has shown that ER stress is involved in I/R injury^19^-^21^. AXL receptor tyrosine kinase AXL is a member of the TAM receptor tyrosine kinases family, which activates numerous critical downstream pathways. Previous studies have showed that AXL reduces inflammation and apoptosis during I/R injury^22^-^25^. However, whether AXL is involved in ER stress pathway, and the detailed mechanism in hepatic I/R injury remains unknown. Herein, we observed that liver I/R increases p-IRE1, XBP1s, p-PERK, ATF4, BiP, CHOP and cleaved-Caspase-12 expression. AXL activation significantly reversed the up-regulation of these ER stress marker proteins during hepatic I/R, indicating that AXL could modulate ER to regain homeostasis. In addition, TEM visually demonstrated the ultrastructure of ER recovered with the up-regulation of activated AXL. As seen in this study, the down-regulation of CHOP by activated AXL inevitably resulted in the down-regulation of apoptotic protein Bax expression and the up-regulation of anti-apoptotic protein Bcl-2 expression, due to the function of CHOP transcribing of these apoptosis-associated proteins^26^.

Mitochondria are directly adjacent to ER, and they maintain the homeostasis of lipids and Ca^2+^ together. The sites where mitochondrial membrane makes contact with ER membrane are called mitochondria-associated ER membranes (MAMs). Recent study has shown organ-to-organ and cell-to-cell crosstalk in the pathogenesis of liver disease^27^, similarly, there are also interactions and functional crosstalk between organelles. The crosstalk between ER and mitochondria plays a key role in cell death, including dynamic changes in intercellular structure and function. Excessive ER stress can induce apoptosis, either by depending or independent on mitochondria^7^, ^28^. In this study, activated AXL inhibited apoptosis by suppressing cleaved-Caspase-9 expression during hepatic I/R injury, and we directly observed *via* TEM that activated AXL not only functionally but also structurally restored the ER and mitochondria, suggesting that activated AXL might induce hepatocytes apoptosis by modulating MAMs to maintain functional transfer between organelles.

Excessive alcohol consumption contributes to the global burden of non-communicable diseases^29^. Increased rates of utilization of steatotic grafts could significantly reduce the current gap between the supply and demand for LT, and previous study revealed that mild steatosis (<30%) affects neither long-term graft function nor patient survival^30^. For patients with early-stage ALD, hepatic steatosis can quickly resolve after complete alcohol withdrawal^31^. This provides a promising theoretical basis for the utilization of donor livers with alcohol-induced steatosis. Although ER stress contributed to ALD development, the molecular mechanisms of regulation in hepatocytes have not been fully elucidated^32^.

Our study experimentally demonstrated that the level of phosphorylated AXL was down-regulated in the hepatocytes of ALD model, which may provide new insights into ALD pathogenesis. Our findings demonstrated that for EtOH-induced steatotic livers, AXL activation can prevent I/R injury by inhibiting ER stress and mitochondria-associated apoptosis. This is a positive sign both for patients with ALD undergoing hepatectomy and the utilization of grafts with mild alcohol-induced steatosis.

In summary, our data demonstrate that AXL activation protects alcoholic fatty liver against ischemia-reperfusion injury by suppressing ER stress and mitochondria-associated apoptosis. These findings suggest that targeting AXL may serve as a promising strategy for hepatic I/R injury, particularly for marginal liver donors with alcohol related steatosis.

## Supplementary Material

Supplementary figures.
