# Supplementary material for: Activated AXL Ameliorates Alcohol-associated Steatotic Liver Ischemia-Reperfusion Injury by Inhibiting ER stress and Mitochondria-associated Apoptosis
Source: Int J Biol Sci. 2025 Jan 20;21(3):1294–307. doi: 10.7150/ijbs.103789 (PMC11781187; doi:10.7150/ijbs.103789)
Supplement: Supplementary file 1 — Supplementary figures. [file ijbsv21p1294s1.pdf]

Fig. S1 Activated AXL attenuates hepatocytes apoptosis during OGD/R *in vitro*. (A-B) WB analysis of the relative protein expression of p-AXL and cleaved-Caspase-3 in primary mouse hepatocytes of control group and treatment group (as shown in the figure) after OGD/R and densitometric quantification. **(C-D)** Flow cytometry assay showing the apoptosis level of primary mouse hepatocytes and corresponding statistical chart. **(E)** TUNEL staining for the level of apoptosis in primary mouse hepatocytes. Scale bar = 100 μm. Data are shown in mean ± SD. *$P < 0.05$, **$P < 0.01$, ***$P < 0.001$; p-AXL, phosphorylated AXL; rmGas6, recombinant growth arrest-specific protein 6; OGD/R, oxygen-glucose deprivation/reoxygenation; TUNEL, Terminal deoxynucleotidyl transferase-mediated nick end labeling.

Fig. S2 (A) Establishment of ALD model and the liver I/R model was conducted. **(B)** Pictures of liver tissues in Pair and ALD mice. **(C)** WB analysis of the relative protein expression of p-AXL in primary hepatocytes with or without EtOH treatment. rmGas6, recombinant growth arrest-specific protein 6; ALD, alcoholic liver disease; p-AXL, phosphorylated AXL; EtOH, ethyl alcohol.

Fig. S1 Activated AXL attenuates hepatocytes apoptosis during OGD/R *in vitro*

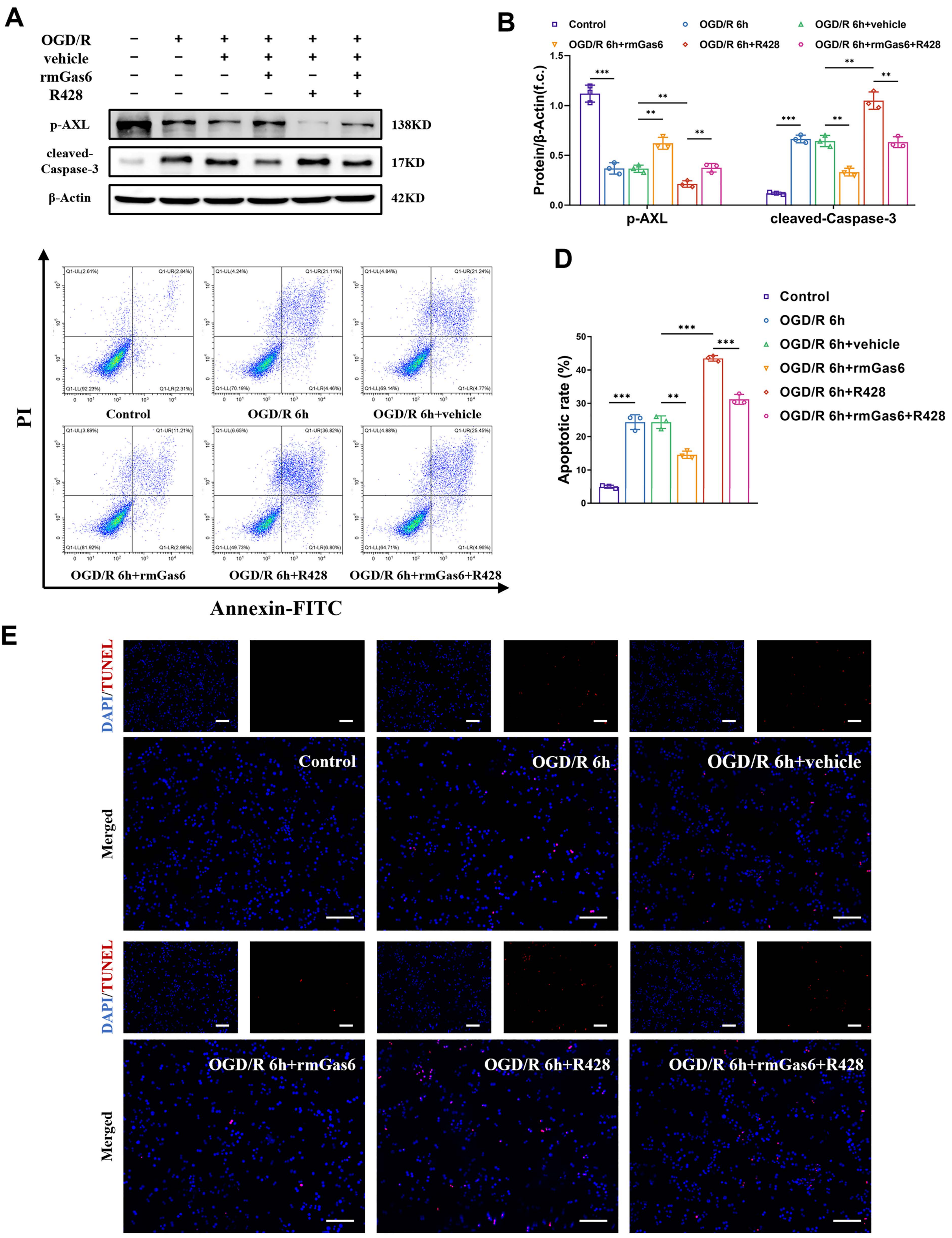

Fig. S2

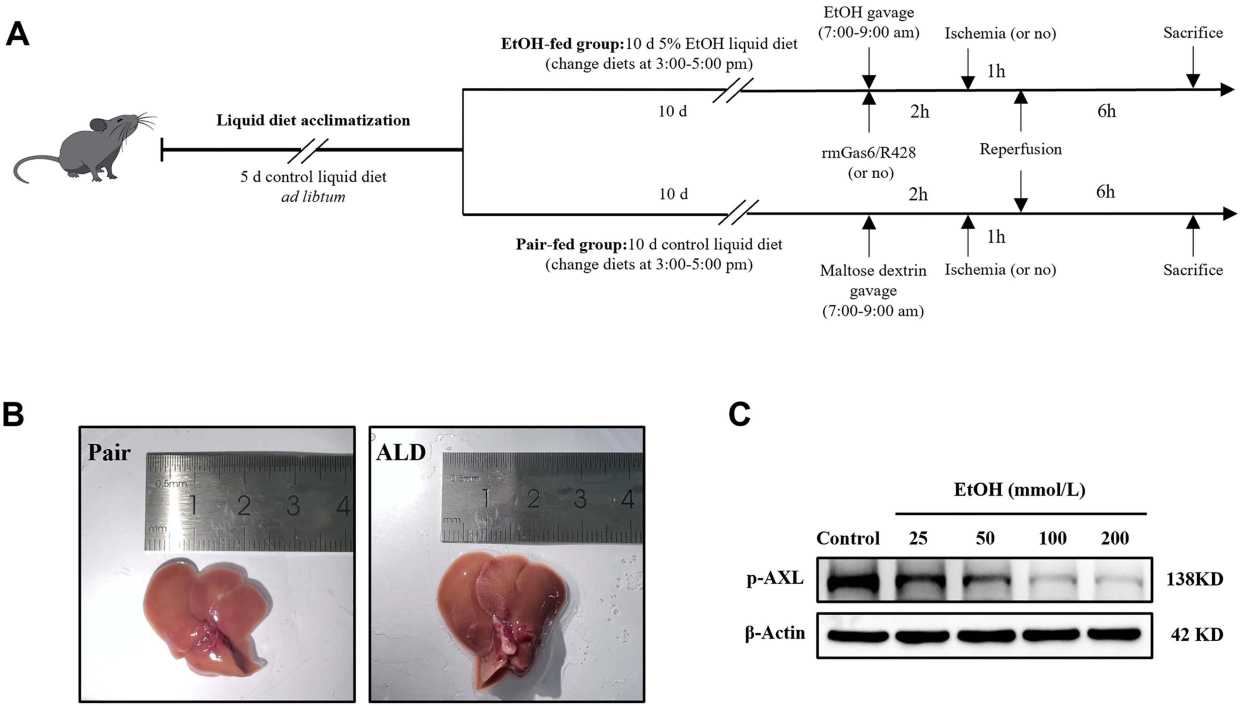
A
EtOH-fed group: 10 d 5% EtOH liquid diet
(change diets at 3:00-5:00 pm)
EtOH gavage
(7:00-9:00 am)
Ischemia (or no)
1h
Sacrifice
10 d
2h
6h
Liquid diet acclimatization
5 d control liquid diet
ad libtum
rmGas6/R428
(or no)
Reperfusion
10 d
2h
6h
1h
Pair-fed group: 10 d control liquid diet
(change diets at 3:00-5:00 pm)
Maltose dextrin
gavage
(7:00-9:00 am)
Ischemia (or no)
Sacrifice
B
Pair
ALD
C
EtOH (mmol/L)
Control
25
50
100
200
p-AXL
138KD
β-Actin
42 KD
